# Validating Reference Gene Expression Stability in Human Ovarian Follicles, Oocytes, Cumulus Cells, Ovarian Medulla, and Ovarian Cortex Tissue

**DOI:** 10.3390/ijms23020886

**Published:** 2022-01-14

**Authors:** Jesús Cadenas, Susanne Elisabeth Pors, Dmitry Nikiforov, Mengxue Zheng, Cristina Subiran, Jane Alrø Bøtkjær, Linn Salto Mamsen, Stine Gry Kristensen, Claus Yding Andersen

**Affiliations:** Laboratory of Reproductive Biology, The Juliane Marie Centre for Women, Children and Reproduction, University Hospital of Copenhagen, Rigshospitalet, 2100 Copenhagen, Denmark; susanne.elisabeth.pors@regionh.dk (S.E.P.); dmitry.embryologist@gmail.com (D.N.); mengxue.zheng@regionh.dk (M.Z.); cristina.subiran.adrados@regionh.dk (C.S.); jane.alroe.boetkjaer@regionh.dk (J.A.B.); linn.salto.mamsen@regionh.dk (L.S.M.); Stine.Gry.Kristensen@regionh.dk (S.G.K.); claus.yding.andersen@regionh.dk (C.Y.A.)

**Keywords:** housekeeping genes, human oocytes, preantral follicles, ovarian tissue, quantitative real-time PCR, NormFinder

## Abstract

Human ovarian cells are phenotypically very different and are often only available in limited amounts. Despite the fact that reference gene (RG) expression stability has been validated in oocytes and other ovarian cells from several animal species, the suitability of a single universal RG in the different human ovarian cells and tissues has not been determined. The present study aimed to validate the expression stability of five of the most used RGs in human oocytes, cumulus cells, preantral follicles, ovarian medulla, and ovarian cortex tissue. The selected genes were glyceraldehyde 3-phosphate dehydrogenase (*GAPDH*), beta-2-microglobulin (*B2M*), large ribosomal protein P0 (*RPLP0*), beta-actin (*ACTB*), and peptidylprolyl isomerase A (*PPIA*). Overall, the stability of all RGs differed among ovarian cell types and tissues. NormFinder identified *ACTB* as the best RG for oocytes and cumulus cells, and *B2M* for medulla tissue and isolated follicles. The combination of two RGs only marginally increased the stability, indicating that using a single validated RG would be sufficient when the available testing material is limited. For the ovarian cortex, depending on culture conditions, *GAPDH* or *ACTB* were found to be the most stable genes. Our results highlight the importance of assessing RGs for each cell type or tissue when performing RT-qPCR analysis.

## 1. Introduction

Human ovarian function is still not completely understood, especially the hormonal mechanisms underlying follicle and oocyte growth and maturation. Understanding these biological processes is particularly relevant in the context of fertility preservation, where procedures such as in vitro follicle culture and oocyte in vitro maturation (IVM) are being developed to augment the outcome of fertility restoration [1,2,3].

The technique of quantitative real-time polymerase chain reaction (RT-qPCR) is considered the benchmark for gene expression analysis and has become the mainstream method to monitor potential improvements during many experimental conditions [4,5,6,7]. Briefly, this method is based on the expression of target genes in relation to endogenous reference genes (RGs), also called housekeeping genes, which are considered to have constant expression. RGs are used to correct sample-to-sample variations, including the amount and integrity of RNA, enzymatic efficiency, and presence of contaminating DNA [8,9]. Ideally, RGs should be universally expressed with a constant level in any cell type and biological or experimental condition [10]. However, no universal RG has been detected so far. Literature shows that RG expression may in fact vary between different tissues, cell types, and experimental conditions [11,12,13,14,15]. Hence, selecting the appropriate RGs is crucial in any experimental design to interpret data generated by RT-qPCR with the best accuracy [16].

Three statistical algorithms have been widely used to assess the stability of RG expression: geNorm [17], BestKeeper [18], and NormFinder [19]. Among them, the NormFinder software is the only one that estimates not only the overall variation of the candidate RGs but also the variation between sample subgroups, which is particularly relevant, for instance, when testing different culture conditions, when a variety of cell types/tissues are involved, or when the samples come from a diverse group of patients.

Despite the fact that RG expression stability has been validated in oocytes and other ovarian cells and tissues from several animal species [20,21,22], no one has, to our knowledge, evaluated human ovarian tissue including oocytes and preantral follicles. However, most studies use a single RG to normalize RT-qPCR data without any preliminary evaluation of appropriateness [4,5,23]. Access to human ovarian tissue is very limited, especially oocytes, and only a few studies have validated RGs in biopsies from normal, cancerous, and/or polycystic ovaries [10,24,25,26], or pieces of cryopreserved vs. non-cryopreserved ovarian cortex [16]. None of these previous studies have investigated RG expression stability in specific ovarian compartments, including oocytes, preantral follicles, cumulus cells, and medulla tissue. Hence, the present study aimed to identify the most stable RGs in order to obtain reliable RT-qPCR results in different compartments of the human ovaries. With this in mind, we selected NormFinder to assess the expression stability of five of the most used RGs in human oocytes, cumulus cells, preantral follicles, ovarian medulla, and ovarian cortex tissue. The RGs included were glyceraldehyde 3-phosphate dehydrogenase (*GAPDH*), beta-2-microglobulin (*B2M*), large ribosomal protein P0 (*RPLP0*), beta-actin (*ACTB*), and peptidylprolyl isomerase A (*PPIA*). Furthermore, the impact of normalizing vs. non-normalizing the RNA concentrations on the RG expression stability was evaluated in oocytes and isolated follicles, and the impact of culture conditions was evaluated in the cryopreserved ovarian cortex.

## 2. Results

### 2.1. General Expression Levels of Reference Genes

Overall, comparisons of the cycle threshold (Ct) values demonstrated a wide variation among the RGs within the same group of samples, especially for oocytes and preantral follicles with normalized RNA (Table 1). ACTB and RPLP0 showed the highest levels of expression and PPIA the lowest levels of expression in all types of samples, except for cortex tissue, where PPIA had the highest level of expression and B2M the lowest (Table 1).

### 2.2. Stability of Reference Genes in Human Oocytes, Preantral Follicles, Cumulus Cells, and Medulla Tissue

The stability values for the five individual RGs and all combinations of RGs were analyzed for each of the different cell types and tissues. NormFinder ranked the RGs, with the most stable RG having the lowest stability value. The mathematical model can be found in the original publication [26].

#### 2.2.1. Ovarian Medulla Tissue

**Figure 1:**
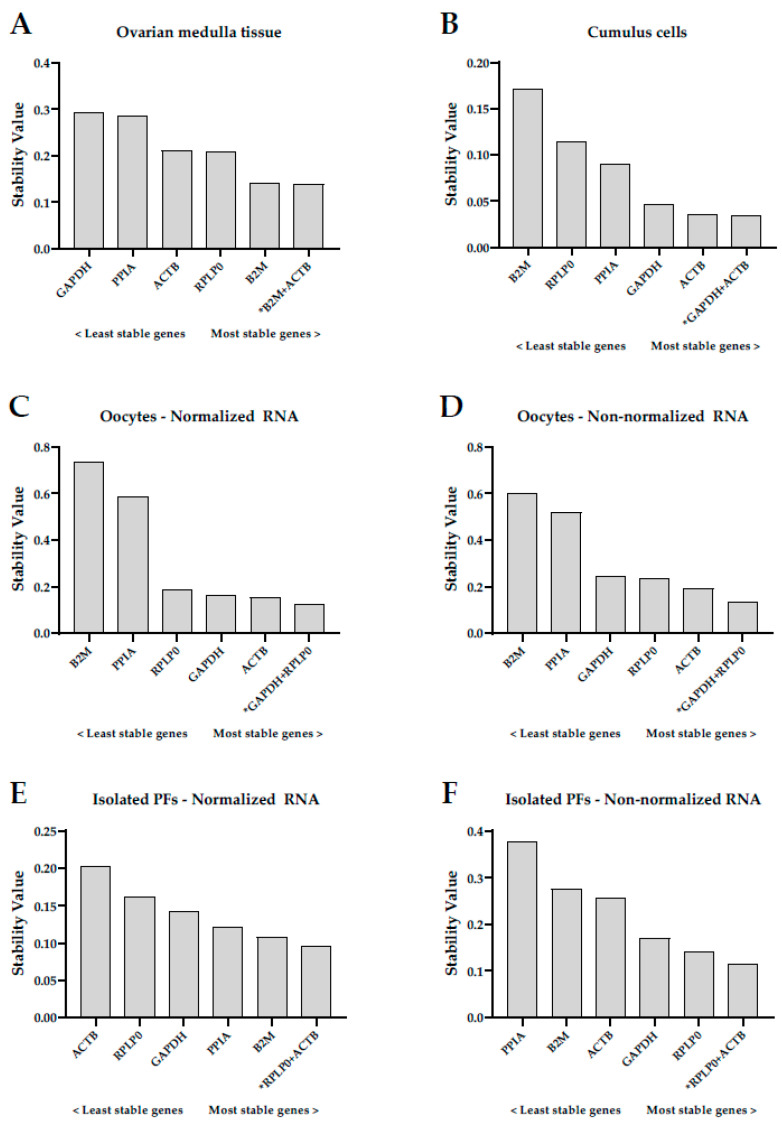
Evaluation of gene expression stability of glyceraldehyde 3-phosphate dehydrogenase (GAPDH), beta-2-microglobulin (B2M), large ribosomal protein P0 (RPLP0), beta-actin (ACTB), and peptidylprolyl isomerase A (PPIA) by the NormFinder software in different human ovarian cells and tissues: (**A**) ovarian medulla tissue; (**B**) cumulus cells; (**C**) oocytes with normalized RNA concentration; (**D**) oocytes with non-normalized RNA concentration; (**E**) isolated preantral follicles (PFs) with normalized RNA concentration; and (**F**) isolated PFs with non-normalized RNA concentration. The genes are ranked from the least (**left**) to the most (**right**) stable. * Best combination of two genes.

Each patient was considered as one subgroup. The ranking of RGs was as follows (starting from the most stable to the least stable): *B2M* > *RPLP0* > *ACTB* > *PPIA* > *GAPDH* (Figure 1A). The best combination of two genes was *B2M* and *ACTB*, with a combined stability value of 0.138, which was very similar to *B2M* alone (stability value = 0.140).

#### 2.2.2. Cumulus Cells

Cumulus cell samples were assessed individually, and each patient was considered as one subgroup. The ranking of RGs was different from that of the medulla tissue, and was as follows (starting from the most stable to the least stable): *ACTB* > *GAPDH* > *PPIA* > *RPLP0* > *B2M* (Figure 1B). The best combination of two genes was *GAPDH* and *ACTB*, with a combined stability value of 0.034, which was similar to *ACTB* alone (stability value = 0.036).

#### 2.2.3. Oocytes

Since each pool of immature GV oocytes contained cells from a large group of patients, the oocyte samples were grouped according to their origin (fresh or IVM) instead of patient. The two arms of this section, i.e., (1) samples with the same RNA concentration (normalized) and (2) samples with different RNA concentrations (non-normalized), had the same number of fresh and IVM oocytes. The ranking of RGs was observed to be similar in both groups, and was as follows (starting from the most stable to the least stable): *ACTB* > *GAPDH* > *RPLP0* > *PPIA* > *B2M* for normalized samples (Figure 1C), and *ACTB* > *RPLP0* > *GAPDH* > *PPIA* > *B2M* for non-normalized samples (Figure 1D). The stability value of *ACTB* alone was 0.154 for normalized samples and 0.191 for non-normalized samples. *GAPDH* and *RPLP0* were selected as the best combination of two genes, with stability values of 0.123 and 0.135 for normalized and non-normalized samples, respectively.

#### 2.2.4. Isolated Preantral Follicles

Each patient was considered as a subgroup when analyzing preantral follicles. Different results were obtained depending on whether the RNA in the samples was normalized or not. The ranking of RGs was as follows (starting from the most stable to the least stable): *B2M* > *PPIA* > *GAPDH* > *RPLP0* > *ACTB* for normalized samples (Figure 1E), and *RPLP0* > *GAPDH* > *ACTB* > *B2M* > *PPIA* for non-normalized samples (Figure 1F). The stability values of the most stable genes were 0.108 (*B2M*) and 0.140 (*RPLP0*) for normalized and non-normalized samples, respectively. The best combination of two genes was *RPLP0* and *ACTB* for both normalized and non-normalized samples, with combined stability values of 0.095 (normalized samples) and 0.115 (non-normalized samples).

### 2.3. Stability of Reference Genes in Human Ovarian Cortex Cultured with Different Serum-Derived Supplements

**Figure 2:**
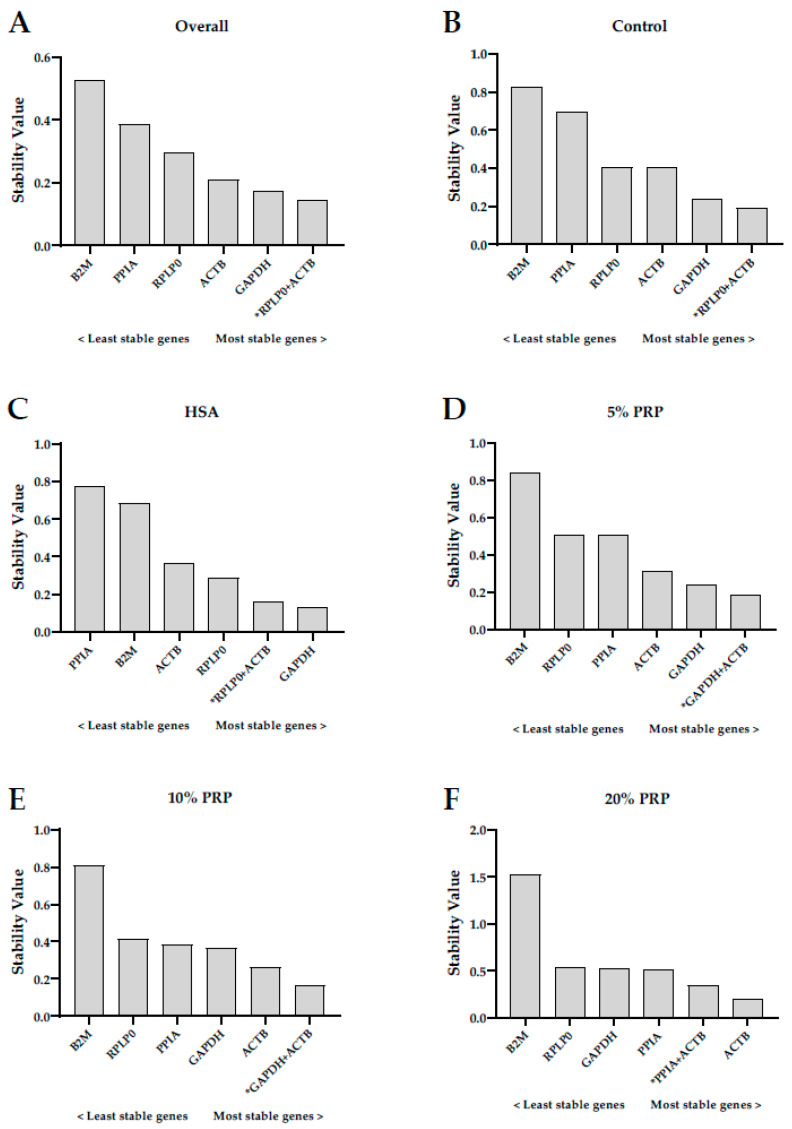
Evaluation of gene expression stability of *GAPDH*, *B2M*, *RPLP0*, *ACTB*, and *PPIA* by the NormFinder software in pieces of ovarian cortex after 24 h incubation with different serum-derived products: (**A**) overall results; (**B**) control treatment; (**C**) culture medium supplemented with human serum albumin (HAS); (**D**–**F**), culture medium supplemented with increasing concentrations of platelet-rich plasma (PRP; (**D**) 5% PRP; (**E**) 10% PRP; and (**F**) 20% PRP). The genes are ranked from the least (**left**) to the most (**right**) stable. * Best combination of two genes.

In a separate experiment, the NormFinder software was used to validate the RG expression stability in the ovarian cortex after a 24 h culture with different serum-derived supplements. For the overall analysis, each culture condition was considered as a subgroup. For the analysis within a particular culture condition, each patient was considered as a subgroup. The overall ranking of RGs in the ovarian cortex after in vitro culture was as follows (starting from the most stable to the least): *GAPDH* > *ACTB* > *RPLP0* > *PPIA* > *B2M* (Figure 2A). The best combination of two genes was *RPLP0* and *ACTB*, with a combined stability value of 0.144 (the stability value of *GAPDH* alone was 0.173). The control treatment showed similar results, with *GAPDH* as the most stable gene, and *RPLP0* and *ACTB* as the best combination of two genes (Figure 2B). Although there were some differences in the rankings of RGs in the HSA and 5% PRP treatments, *GAPDH* was also selected as the most stable gene in both treatments, with stability values of 0.131 and 0.242 for HSA and 5% PRP, respectively (Figure 2C,D). The best combinations of two genes were *RPLP0* and *ACTB* for the HSA treatment (stability value = 0.162), and *GAPDH* and *ACTB* for the 5% PRP treatment (stability value = 0.187). Unlike the other treatments, *ACTB* and not *GAPDH* was selected as the most stable gene when the concentrations of PRP were 10% or 20%, with stability values of 0.264 and 0.194, respectively (Figure 2D,F). Here, the best combinations of two genes were *GAPDH* and *ACTB* for 10% PRP (stability value = 0.166), and *PPIA* and *ACTB* for 20% PRP (stability value = 0.342).

## 3. Discussion

Overall, the present study demonstrates that the RG expression stability differed according to the type of human ovarian cells and tissues and is furthermore affected by culture conditions. The NormFinder tool found *ACTB* to be the most stable gene for human immature oocytes and cumulus cells, and *B2M* to be the most stable gene for medulla tissue and isolated preantral follicles, provided a normalized RNA concentration was used. Even though *ACTB* is one of the most used RGs, some studies omit this as RG because its expression can be affected by biochemical stimuli, during cell growth and differentiation, or even by certain pathologies in various human cells and tissues [12], including ovarian tissue [10]. Nevertheless, our data suggest that *ACTB* expression in human immature oocytes is more stable than in other cell types as we obtained similar results with both fresh and in vitro cultured immature oocytes (data not shown). None of the ovaries included in this study had any apparent pathology, and all oocytes were at the same maturation stage (germinal vesicle, GV). Therefore, it is beyond the competence of this study to address whether ovarian pathologies or other maturational stages (e.g., metaphase II oocytes) affect *ACTB* expression.

In the case of *B2M*, there are no available data about its use as RG for ovarian medulla tissue or isolated follicles in any species. However, in bovine, *B2M* has been regarded as a stable RG for different follicle cell types, such as granulosa cells after culture [27], as well as oocytes and cumulus cells after IVM [22].

The combinations of *RPLP0* and *GAPDH* for oocytes, along with *RPLP0* and *ACTB* for follicles, provided marginally better stability values than *ACTB* and *B2M* alone. However, none of the cells and tissues showed marked improved stability scores by combining either one of the RGs. Nevertheless, it may be prudent to use two RGs in situations where the RNA availability is not an issue or where many treatments are tested. The principle is simple; the mean variation in the expression levels of multiple genes is smaller than in single genes [19]. Despite that, the use of more than one RG does not necessarily imply an improved expression stability. Hence, the number of genes to be included as endogenous controls should be carefully considered; the number of genes should be a balance between practical considerations and minimizing gene expression variation. For human oocytes, which are a particularly precious material that provides a very limited amount of RNA, it can be recommended to use a single validated RG, which, in many situations, may be considered sufficient.

Normalizing RNA concentration before reverse transcription, i.e., loading the same RNA concentration in all samples, is common practice in RT-qPCR. Nonetheless, the quantification of target gene expression in a single RNA sample is relative to the expression of one or more RGs in that specific sample. Therefore, for each sample, the ratio of a target gene to RG should be consistent, and then, the quantity of RNA added, at least in theory, is not critical. Nevertheless, it turns out that the efficiency of reverse transcription depends, among other things, on the total RNA concentration [28]. Accordingly, different RGs were selected as the most stable gene for isolated follicles when the RNA concentrations were normalized vs. non-normalized: *B2M* and *RPLP0* for normalized and non-normalized, respectively. Interestingly, RNA normalization did not affect RG expression stability in oocytes, with *ACTB* being ranked as the most stable gene in both normalized and non-normalized samples. This may be due to the fact that the amount of RNA extracted from pools of 10 denuded oocytes is very similar, unlike isolated follicles, where it is not possible to know exactly the number of cells loaded within each 10-follicle pool. Interestingly, the same best combinations of two genes were selected for normalized and non-normalized samples. Hence, using two RGs could be a good strategy, especially when RNA concentrations cannot be normalized.

We also found that reference gene expression stability was affected by culture conditions in the human ovarian cortex, highlighting that cell manipulation may also impact gene expression. Overall, *GAPDH* was the most stable gene for this type of tissue. *GAPDH* was the most stable gene in the control, HSA, and 5% PRP treatments, whereas *ACTB* was the most stable gene for 10% and 20% PRP. Our results are comparable to the effect of how serum supplementation affects RG expression during culture in colon adenocarcinomas cell lines [15]. A few studies have also validated RG expression stability in biopsies from normal, cancerous, and polycystic ovaries (PCO) [10,24,25,26] or pieces of cryopreserved versus the non-cryopreserved ovarian cortex [16], with variable outcomes. *GAPDH* has proven to be stably expressed in normal ovarian tissues [24], whereas both *GAPDH* and *ACTB* have been poorly ranked in PCO and cancerous ovarian tissue [10,25,26]. It is worth noting that, in our study, the patient effect was considered within each treatment.

The present study has some limitations. The NormFinder tool demands the availability of a considerable amount of RNA; it requires a minimum of three genes and eight samples per group to perform the analysis [26]. Since human oocytes are scarce and small and only contain a modest amount of RNA, only five of the most commonly used RGs were evaluated. Therefore, it cannot be excluded that there can be other, more stable genes. Moreover, we only included immature oocytes and preantral follicles of around 60–80 µm in diameter. In addition, a limiting factor of the present study is that the hormonal status, age, and medical condition of each patient may affect gene expression. The components of the human ovary are only available in very limited supply, and if material from more patients can be collected, additional studies to investigate these conditions are warranted.

In conclusion, here we present the most complete evaluation of RG expression stability to date in human ovarian tissues and cells, including oocytes and preantral follicles. Our findings highlight the importance of assessing and validating RGs for each cell type or tissue to choose the most stable RGs for RT-qPCR. Moreover, for some cell types, the combination of two RGs only provided a modest increment of the stability value, which indicated that using a single validated RG can be recommended when the available testing material is limited. Hence, in our study conditions, we suggest using *ACTB* for oocytes and cumulus cells, and *B2M* for medulla tissue and isolated follicles. For the ovarian cortex, culture conditions affected the RG expression stability; depending on the treatment, *GAPDH* or *ACTB* were found to be the most stable genes.

## 4. Materials and Methods

### 4.1. Experimental Design

**Figure 3:**
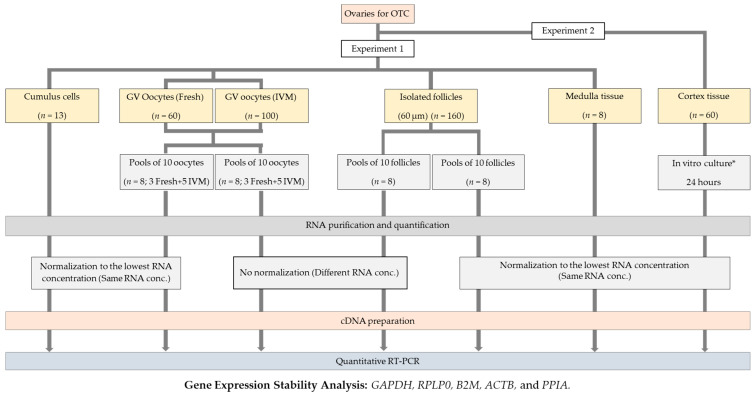
Experimental design. The stability of *GAPDH*, *B2M*, *RPLP0*, *ACTB*, and *PPIA* was analyzed in different human ovarian cells and tissues by the NormFinder software. Two experiments were performed: In experiment 1, gene expression stability of the five RGs was analyzed in human oocytes, isolated preantral follicles, cumulus cells, and ovarian medulla tissue. In experiment 2, the stability of the five RGs was assessed in pieces of ovarian cortex after 24 h of culture in vitro under different conditions. Oocytes and follicles were subdivided into two groups depending on whether the RNA concentrations before cDNA synthesis were normalized or not, i.e., half of the samples had the same RNA concentration converted into cDNA (normalized), and the other half had a different RNA concentration within each sample converted into cDNA (not-normalized). The RNA concentrations in cumulus cells, ovarian medulla tissue, and ovarian cortex were all normalized. * Culture conditions for human ovarian cortex: base medium alone (Control treatment), base medium supplemented with 5 mg/mL HSA (HSA treatment), or base medium supplemented with increasing concentrations of PRP (5% PRP, 10% PRP, and 20% PRP treatments).

The experimental design is shown in Figure 3. The stability of five of the most commonly used reference genes (RGs) (i.e., *GAPDH*, *B2M*, *RPLP0*, *ACTB*, and *PPIA*) was analyzed by the NormFinder software. The study had two arms assessing either the stability of RGs in isolated ovarian cells and tissues or under different culture conditions. In experiment 1, the gene expression stability of the five RGs was analyzed in human oocytes, isolated preantral follicles, cumulus cells, and ovarian medulla tissue. In experiment 2, the stability of the five RGs was assessed in pieces of ovarian cortex after 24 h of culture.

Within experiment 1, oocytes and follicles were subdivided into two groups depending on whether the RNA concentrations before cDNA synthesis were normalized or not, i.e., half of the samples had the same RNA concentration converted into cDNA (normalized), and the other half had a different RNA concentration within each sample converted into cDNA (non-normalized). The RNA concentrations in cumulus cells, ovarian medulla tissue, and ovarian cortex were all normalized.

### 4.2. Patients and Collected Material

A total of 29 patients (aged 28 years on average; range 14–36) undergoing unilateral oophorectomy and OTC for fertility preservation [29] were included in the study. None of the patients presented with pathologies in the ovaries. The indications for fertility preservation were Hodgkin lymphoma (*n* = 3), non-Hodgkin lymphoma (*n* = 1), breast cancer (*n* = 17), sarcoma (*n* = 3), and various benign diseases (*n* = 5). All samples used in the present study (oocytes, cumulus cells, isolated preantral follicles, ovarian medulla tissue, and ovarian cortex) were collected in association with other research projects, i.e., only a fraction of the samples collected from a single patient was allocated to this study. The type of sample used from each patient is shown in Supplementary Table S1. In experiment 1, immature oocytes (*n* = 160) at germinal vesicle (GV) stage were collected from 22 patients, including 60 fresh oocytes (Fresh-GV) from 7 patients, and 100 oocytes after IVM (GV-IVM) from 15 patients; preantral follicles (*n* = 160) from 7 patients; cumulus cells (*n* = 13) from 3 patients; and medulla tissue (*n* = 8) from 4 patients. In experiment 2, sixty pieces (2 × 2 × 2 mm) of ovarian cortex were cultured from 4 patients (15 pieces of ovarian tissue from each patient). The fertility history of the patients and the phase of their menstrual cycle were not recorded.

### 4.3. Ovary Transport and Oocyte Collection

Ovaries were transported to the laboratory in IVF flushing medium (Origio A/S, Måløv, Denmark) or in Custodiol^®^ HTK (Dr Franz Köhler Chemie GmbH., Bensheim, Germany), either on crushed ice from collaborating hospitals (2–5 h transport) or at 37 °C from the local hospital (10 min transport) [30]. After isolating the ovarian cortex for cryopreservation, dishes containing the surplus medulla tissue in HEPES-buffered DMEM/F-12 medium (GIBCOTM, Life Technologies, Paisley, UK) were thoroughly examined for the presence of immature oocytes under a stereomicroscope (Leica MZ12, Wetzlar, Germany) within a flow hood with heated tabletop at 37 °C. Recovered oocytes were placed in oocyte holding medium, which consisted of McCoy’s 5α plus 25 mM HEPES (Invitrogen, GIBCOTM) with 5 mg/mL human serum albumin (HSA; 20%, CSL Behring, Marburg, Germany), 10 µg/mL insulin, 5.5 µg/mL transferrin, 6.7 ng/mL selenium (ITS; GIBCOTM, Life Technologies, Grand Island, NY, USA), 2 mM Glutamax (GIBCOTM, Life Technologies, Paisley, UK), and 0.05 mg/mL penicillin/streptomycin (GIBCOTM, Life Technologies, Paisley, UK) [6]. After that, cumulus-enclosed oocytes were mechanically denuded using a 130–133 µm denudation pipette (Vitrolife, Gothenburg, Sweden) and visualized under an inverted microscope (Carl Zeiss Axiovert 135, Wetzlar, Germany; 20× magnification). Only oocytes that showed clear signs of degeneration, such as darkened cytoplasm, were excluded from the study. Selected immature oocytes at GV stage were then washed in Dulbecco’s phosphate buffered saline (DPBS; GIBCOTM, Life Technologies, Paisley, UK), pooled in groups of 10, snap frozen, and stored at −80 °C (GV-Fresh, *n* = 60).

In combination with another project, oocytes that remained immature, i.e., at the GV stage after IVM, were also denuded, pooled in groups of 10, snap frozen, and stored at −80 °C (GV-IVM, *n* = 100).

### 4.4. Medulla Tissue Collection and Follicle Isolation

Eight pieces of ovarian medulla tissue were snap frozen and stored at −80 °C. The remaining pieces of surplus medulla tissue were used for follicle isolation as previously described, with some modifications [31]. Briefly, all medulla tissue pieces were collected in HEPES-buffered DMEM/F-12 medium and mechanically minced into fragments with a McIlway tissue chopper (Cavey Laboratory Engineering Co. Ltd., Guildford, Surrey, UK). Tissue pieces were transferred to a 100 mm culture dish (Nunclon^TM^ Delta, Nunc A/S, Roskilde, Denmark) containing preheated follicle holding medium that consisted of McCoy’s 5α plus 25 mM HEPES with 1 mg/mL HSA, 1% ITS, 2 mM Glutamax, and 0.05 mg/mL penicillin/streptomycin. For tissue enzymatic digestion, the holding medium was supplemented with 0.2 mg/mL collagenase IV (Sigma-Aldrich, Saint Louis, MO, USA) and 0.2 mg/mL DNase I (Sigma-Aldrich, USA), followed by incubation at 37 °C for 60 min with gentle agitation. The enzymatic digestion was terminated by adding an equal volume of McCoy’s 5α plus 25 mM HEPES with 10% fetal bovine serum (FBS, Invitrogen, GIBCO^TM^), and 0.05 mg/mL penicillin/streptomycin. The cell suspension was vigorously aspirated up and down with a 3 mL Pasteur pipette (Vitrolife, Sweden) to release the follicles from the digested tissue. Culture dishes were carefully examined for the presence of preantral follicles under a stereomicroscope (Leica MZ12, Wetzlar, Germany) at 37 °C. Recovered preantral follicles were placed in follicle holding medium and visualized under an inverted microscope connected to a camera (DeltaPix, Smørum, Denmark) to check basement membrane integrity and diameter. Follicles that were around 60–80 µm in diameter were pooled in groups of 10 (*n* = 160), washed in DPBS, snap frozen, and stored at −80 °C. Follicles with larger diameters were used in a different study.

### 4.5. In Vitro Culture of Human Ovarian Cortex (Experiment 2)

Four pieces of ovarian cortex (5 × 5 × 2 mm) from four patients that had been previously cryopreserved and donated for research were used. Each piece was thawed and cut into fifteen fragments (*n* = 60); from those, three fragments were allocated to each of the five treatment groups: base medium alone, consisting of α-minimum essential medium (α-MEM; GIBCO^TM^, Life Technologies, Bleiswijk, The Netherlands) with 1% ITS, 5 IU/mL heparin (Amgros I/S, Copenhagen, Denmark) and 0.05 mg/mL penicillin/streptomycin (control treatment), base medium supplemented with 5 mg/mL HSA (HSA treatment), or base medium supplemented with increasing concentrations of platelet-rich plasma (PRP) (5% PRP, 10% PRP, and 20% PRP). PRP was prepared and kindly donated by the blood bank of the University Hospital of Copenhagen, Rigshospitalet, Denmark. Each fragment was transferred into an individual well of a 48-well plate containing 300 µL of culture medium at 37 °C and 5% CO_2_ for 24 h. After that, the ovarian fragments were snap frozen and stored at −80 °C.

### 4.6. Quantitative Real-Time PCR (RT-qPCR)

Different human ovarian tissues were evaluated in two experiments: medulla tissue (*n* = 8), immature oocytes (*n* = 16 pools, 10 oocytes per pool), cumulus cells (*n* = 13), and isolated preantral follicles (*n* = 16 pool, 10 follicles per pool) (Experiment 1); and ovarian cortex (*n* = 60) after in vitro culture (Experiment 2). Total RNA was extracted and purified from each sample with TRIzol^®^ reagent (Ambion, Life Technologies, Carlsbad, CA, USA) and 1-bromo-3-chloropropane (Sigma-Aldrich, USA) and, subsequently, with RNeasy^®^ Minikit 250 (Qiagen, Denmark) according to manufacturer’s instructions. All steps were performed on ice. The quality and quantity of the isolated RNA was evaluated using Agilent RNA 6000 Pico kit and Agilent Bioanalyzer 2100 (Agilent Technologies, Santa Clara, CA, USA). Only samples with an RNA integrity value (RIN) ≥ 5 were included in the study. For each selected sample, first-strand cDNA was prepared using the High Capacity cDNA Reverse Transcription Kit (Applied Biosystems, Foster City, CA, USA) following the manufacturer’s instructions. The quantitative RT-PCR analysis was carried out by TaqMan^®^ technology using the TaqMan^TM^ Fast Advanced Master Mix (Applied Biosystems, Foster City, CA, USA). The specific TaqMan probes used in the study are shown in Table 2. For each gene, the TaqMan probe with the best coverage was selected based on the criteria and as recommended by the manufacturer (Thermo Fisher Scientific). According to information from the manufacturer, amplification efficiency was 100% ± 10%. All samples were run in duplicates and two negative controls (a non-template RNA RT–PCR control and a non-template cDNA control) were prepared according to manufacturer’s instructions. The absolute expression levels were quantified using the LightCycler480 Software (Roche, Basel, Switzerland).

### 4.7. Statistical Analysis

For stability comparisons of RGs, the NormFinder algorithm was used (v0.953) according to its original publication [26]. The mRNA expression level for each of the 5 RGs was obtained using the mean RT-PCR Ct-value (cycle threshold), which is defined as the number of cycles needed for the fluorescence to exceed background levels, and is inversely proportional to the amount of target nucleic acid present in the reaction. The Ct-values were transformed to linear scale expression quantities by 2^−Ct^ and exported to NormFinder for analysis. Comparison of the Ct-values for different genes was carried out using a linear mixed model, with Ct as outcome, gene as explanatory variable and patient as random effect when the samples were not pooled. The linear model resulted in a Wald test. Log transformation of data was used when not normal distributed. All analyses were performed in R, v3.4.3.

## Figures and Tables

**Table 1:** Mean Ct values ± SD (% deviation) of the studied reference genes in each of the different samples from human ovaries.

	Preantral Follicles		Oocytes		Cumulus Cells	Medulla Tissue	Cortex Tissue
RGs	Normalized	Non-Normalized	Normalized	Non-Normalized	Normalized	Normalized	Normalized
*GAPDH*	30.1 ± 0.5 ^a^ (1.6%)	29.7 ± 1.1 ^a^ (3.7%)	30.0 ± 2.2 ^a^ (7.3%)	30.4 ± 2.5 ^a^ (8.2%)	25.6 ± 1.2 ^a^ (4.7%)	23.6 ± 1.5 ^a^ (6.4%)	21.6 ± 1.0 ^a^ (4.6%)
*RPLP0*	27.7 ± 0.4 ^b^ (1.4%)	27.6 ± 1.0 ^b^ (3.6%)	28.2 ± 2.4 ^b^ (8.5%)	28.7 ± 2.3 ^b^ (8.0%)	24.1 ± 1.1 ^b^ (4.6%)	20.9 ± 1.6 ^bc^ (7.4%)	21.3 ± 0.7 ^a^ (3.3%)
*B2M*	29.2 ± 0.6 ^c^ (2.0%)	29.3 ± 1.6 ^a^ (5.5%)	31.3 ± 3.3 ^c^ (10.5%)	33.1 ± 3.1 ^c^ (9.4%)	26.3 ± 1.3 ^a^ (4.9%)	21.6 ± 1.6 ^c^ (7.4%)	25.6 ± 2.5 ^b^ (9.8%)
*ACTB*	26.7 ± 0.8 ^d^ (3.0%)	26.7 ± 1.5 ^c^ (5.6%)	26.9 ± 2.2 ^d^ (8.2%)	28.1 ± 2.4 ^b^ (8.5%)	22.5 ± 1.1 ^c^ (4.9%)	20.6 ± 1.3 ^b^ (6.3%)	23.4 ± 0.7 ^a^ (3.0%)
*PPIA*	34.1 ± 0.4 ^e^ (1.2%)	33.5 ± 0.8 ^d^ (2.4%)	32.7 ± 1.6 ^e^ (4.9%)	33.3 ± 1.7 ^c^ (5.1%)	29.4 ± 1.5 ^d^ (5.1%)	27.1 ± 1.7 ^d^ (6.3%)	21.2 ± 1.4 ^c^ (6.6%)

RGs: Reference genes. Normalized: The RNA concentration was adjusted—All samples contained the same RNA concentration within a sample type. Non-normalized: The RNA concentration was not adjusted—All samples contained different RNA concentrations within a sample type. Comparison of the Ct-values for different genes was carried out using a linear mixed model with Ct as outcome, gene as explanatory variable, and patient as random effect when the samples were not pooled. The linear model results in a Wald test. Log transformation of data was used when not normal distributed. Within a column, different lowercase letter (^a–e^) indicates *p* ≤ 0.006.

**Table 2:** Candidate reference genes used in the study.

Gene Symbol	Gene Name	TaqMan Probe
*GAPDH*	Glyceraldehyde 3-phosphate dehydrogenase	Hs02786624_g1
*ACTB*	Beta-actin	Hs01060665_g1
*RPLP0*	Large ribosomal protein P0	Hs00420895_gH
*B2M*	Beta-2-microglobulin	Hs00187842_m1
*PPIA*	Peptidylprolyl isomerase A	Hs04194521_s1

## Data Availability

All data are available via the corresponding author.

## Supplementary Materials

The following are available online at https://www.mdpi.com/article/10.3390/ijms23020886/s1.
