# Supplementary material for: Validating Reference Gene Expression Stability in Human Ovarian Follicles, Oocytes, Cumulus Cells, Ovarian Medulla, and Ovarian Cortex Tissue
Source: Int J Mol Sci. 2022 Jan 14;23(2):886. doi: 10.3390/ijms23020886 (PMC8778884; doi:10.3390/ijms23020886)
Supplement: Supplementary file 1 [file ijms-23-00886-s001.zip › ijms-1536947-supplementary.pdf]

Table S1. Type of sample collected from each patient included in the study

Patient	Sample type Oocytes (GV-Fresh)	Oocytes (GV-IVM)	Preantral follicles	Cumulus cells	Medulla tissue	Cortex tissue
1	*					
2	*		*			
3	*		*			
4	*		*	*		
5			*			
6	*			*		
7			*		*	
8	*				*	
9			*		*	
10	*		*	*	*	
11		*				
12		*				
13		*				
14		*				
15		*				
16		*				
17		*				
18		*				
19		*				
20		*				
21		*				
22		*				
23		*				
24		*				
25		*				
26						*
27						*
28						*
29						*

GV-Fresh, non-cultured immature oocytes collected at the germinal vesicle stage. GV-IVM, oocytes that remained at the GV stage after in vitro maturation (IVM). Preantral follicles were around 60 μm in diameter.
